# The tumor biological significance of RNF43 and LRP1B in gastric cancer is complex and context-dependent

**DOI:** 10.1038/s41598-023-30294-8

**Published:** 2023-02-23

**Authors:** Bente Holm, Stephan Barsuhn, Hans-Michael Behrens, Sandra Krüger, Christoph Röcken

**Affiliations:** 1 Department of Pathology, Christian-Albrechts-University, Kiel, Germany; 2 Department of Pathology, Christian-Albrechts-University, University Hospital Schleswig-Holstein, Arnold-Heller-Str. 3, Haus 14, 24105 Kiel, Germany

**Keywords:** Biomarkers, Gastroenterology, Oncology

## Abstract

Gastric cancer (GC) is the fifth most common cancer in the world with a poor prognosis. Both RNF43 and LRP1B function as tumor suppressors in the Wnt signaling pathway and have been described to be frequently mutated in GC. In this study of a large and well characterized cohort of 446 GCs we explored the significance of expression of RNF43 and LRP1B and their correlations with clinicopathological patient characteristics. Immunostaining of whole mount tissue sections was documented with the histoscore. Dichotomized at the median, we separated the cohort into a low/negative and a high/positive group of RNF43 and LRP1B expression, respectively. Apart from the entire cohort, we also examined the intestinal and diffuse type GCs separately. Regarding the entire cohort, the expression of RNF43 and LRP1B correlated significantly with the Lauren phenotype and with each other. Interestingly, differences were noted regarding RNF43 between the intestinal and diffuse type GCs. Survival analysis of the intestinal type GCs showed that RNF43 low/negative GCs tended to have a better outcome compared with RNF43 high/positive GCs [24.5 months overall survival (OS) and 25.0 months tumor-specific survival (TSS) vs. 14.1 months OS and 17.9 months TSS, respectively]. To the contrary, diffuse type GCs with RNF43 low/negative had a worse outcome compared with RNF43 high/positive GCs (12.9 months OS and 18.2 months TSS vs. 17.1 months OS and 21.5 months TSS, respectively). On multivariate analysis, RNF43 low/negative versus high/positive was an independent prognosticator of survival in diffuse type GC (hazard ratio 2.393 for OS and 2.398 for TSS). These data support the contention that the expression and biological effect of RNF43 and LRP1B in GC is context-dependent.

## Introduction

Gastric adenocarcinoma (GC) is the fifth most common cancer and the third leading cause of cancer related deaths worldwide. The etiology is diverse, and known risk factors are the colonization of the stomach’s mucosa by Helicobacter pylori, the consumption of tobacco and a diet rich in salt^1^. Histologically, GC is most commonly classified according to Lauren^2^. More recently, a molecular classification distinguishing four subtypes was proposed, i.e., Epstein–Barr-virus positive, microsatellite instable, chromosomal instable, and genomically stable GC^3^. Furthermore, GC is characterized by intra- and intertumoral heterogeneity, where different tumor subclones coexist and contribute to genetic and phenotypic diversity. Using multiregional whole exome sequencing, we recently investigated the effect of somatic evolution on intratumoral heterogeneity aiming to shed light on the evolutionary biology of GC and noted that *RNF43* and *LRP1B* are the among the most commonly mutated genes in GC^4^.

Ring Finger Protein 43 (RNF43) is an integral membrane protein playing an important role in the Wnt signaling pathway. The Wnt signaling pathway is involved in the development and homeostasis of different tissue types and plays a key role in initiation and development of cancer. In GC, mutations in the Wnt signaling pathway are frequently found^5^. Particularly, *RNF43* has been established as a tumor suppressor gene, negatively regulating the Wnt signaling pathway and mutations of *RNF43* occur in as many as 3–44% of GCs^6^. Previous investigations showed that reduced RNF43 expression in tumor cells is associated with increased cell proliferation and increased invasive capacity^7,8^, and reduced or lost expression of RNF43 predicts poor survival^9^.

The low-density lipoprotein receptor-related protein 1b (LRP1B) is a recently discovered member of the low-density lipoprotein receptor family. Like RNF43, the LDL receptor family is also involved in the Wnt signaling pathway. They are widely expressed in nearly all cells and their biological functions are highly diverse including cell signaling, gene regulation and cargo transport^10^. In GC, *LRP1B* is mutated in 31–67% of the cases^11^. It is also regarded as a tumor suppressor of the Wnt signaling pathway playing an important role in cancer development^12^. Thus, both, *RNF43* and *LRP1B* are among the most commonly mutated genes in GC underscoring their putative significance in cancer development and progression.

**Figure 1:**
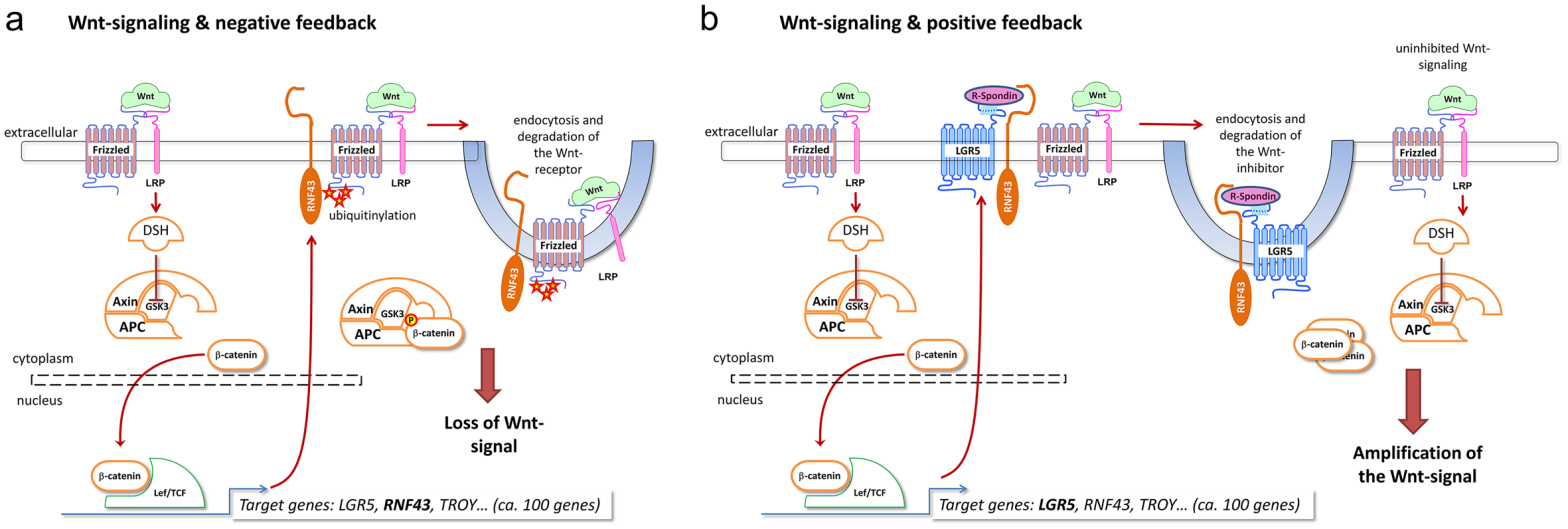
Wnt signaling pathway and its regulatory mechanisms. Wnt ligand binds to FZD-LRP5/6 complex. DVL inhibits the destruction complex (DC) so that β-catenin is not degraded but accumulates in the cytoplasm, translocates into the nucleus and activates transcription factors. RNF43 and LRP1B act as negative regulators of the Wnt signaling pathway (**a**). Mutations in RNF43 or LRP1B or their loss lead to increased Wnt signaling (**b**).

However, to understand the Wnt signaling pathway with its negative or positive feedback mechanisms, it is decisive to become aware of the interaction between all molecules playing an important role in this pathway (Fig. 1). The crucial signaling molecule is β-catenin. Its stability is controlled by the destruction complex (DC) consisting of Axin, APC and GSK3. If no Wnt ligand binds to the FZD-LRP5/6-receptor complex, β-catenin is ubiquitinated and degraded by the proteasome. During activation of the FZD-LRP5/6 complex by a Wnt ligand, dishevelled (DVL) leads to inhibition of DC. Hereinafter, the β-catenin-level increases and accumulates in the cytoplasm eventually translocating into the nucleus. β-catenin forms a protein complex together with the T-cell factor/lymphoid enhancer factor (TCF/LEF) and thus activates specific target genes^13^, such as *RNF43*. RNF43 ubiquitinates FZD and induces endocytosis and degradation of the Wnt receptor^14^. This leads to a loss of the Wnt-signal and RNF43 regulates this pathway via negative feedback mechanism. LRP1B interacts with DVL2 inhibiting the β-catenin/TCF signaling^12^. Consequently, mutations of *RNF43/LRP1B* and/or loss of expression of RNF43/LRP1B often end up in a dysfunction of the negative feedback and thereby in an increased Wnt-signaling.

However, despite their putative tumor biological role, data on the expression of RNF43 and LRP1B in GC are scarce. In order to fill this gap of information, we explored the tumor biological significance of RNF43 and LRP1B in a large and extensively characterized cohort of therapy-naive GC by immunohistochemistry. In this study we tested the following hypotheses: (1) RNF43 and LRP1B are of tumor biological significance in GC; (2) RNF43 and LRP1B show intratumoral heterogeneity; (3) RNF43-expression and LRP1B-expression correlate with clinicopathological patient characteristics and show differences with regard to histological type of Lauren.

## Methods

### Ethics statement

This study was performed according to the Declaration of Helsinki. Tissue samples originated from routine therapeutic surgeries, for all of which the patients had given written informed consent. Ethical approval was obtained from the local ethical review board (D 453/10 and D 525/15) of the University Hospital Schleswig–Holstein, Kiel, Germany, which permitted us to use the samples from those patients, who had also given written informed consent for a prospective scientific use of their patient material^15^.

### Study populations

#### Discovery group

For the discovery group, we prospectively enrolled between 2016 and 2017 nine patients with an adenocarcinoma of the stomach or esophagogastric junction at the University Hospital Schleswig–Holstein, Campus Kiel. All patients were Caucasian patients from Northern Germany treated in a single center. The mean age of the patients were 68 years and the age ranged from 50 to 85 years. The inclusion criteria were appropriate size of the primary tumor (diameter > 3 cm) to enable multiregional tissue sampling without compromising the surgical pathological evaluation of the resection specimen. After the tumor was resected, the specimens were delivered on ice to the Department of Pathology. Depending on the size of the primary tumor, between 3 and 10 samples were punched out of the primary tumor using a core needle biopsy and frozen at − 80 °C until further use. Macroscopic pictures were taken from the surgical resection before and after tissue sampling in order to facilitate anatomical reconstruction of the sampling procedure and correlation with paraffin blocks. A total of 44 samples were obtained from the primary tumors. In a single case, three samples were collected from three separate lymph node metastases. Finally, in total 47 tumor samples were forwarded to whole-exome sequencing (WES). The detailed genomic data of this cohort were described previously^4^.

#### Validation group

From the archive of the Department of Pathology, University Hospital Schleswig–Holstein, Campus Kiel we retrieved all patients who had undergone either total or partial gastrectomy for an adenocarcinoma of the stomach or gastroesophageal junction between 1997 and 2009. The following patient characteristics were retrieved: age at diagnosis, gender, tumor size, tumor localization, tumor type, tumor grade, depth of tumor invasion, number of lymph nodes resected and number of lymph nodes with metastases, distant metastases, stage of disease, invasion into lymphatic vessels and into veins and residual tumor status. Patients were included if a primary adenocarcinoma of the stomach or gastroesophageal junction was histologically confirmed. Patients were excluded if the histology identified a tumor type other than adenocarcinoma, if patients had undergone perioperative chemo- or radiotherapy or if the tumor had developed in the residual stomach after Billroth-resection. Each resected specimen had undergone histological examination by trained and board certified surgical pathologists. The data of patient death were obtained from the Epidemiological Cancer Registry of the state of Schleswig–Holstein, Germany. Follow-up data of the patients who were still alive were retrieved from hospital records and general practitioners. All patient data were pseudonymized after inclusion in the study^16^.

### Histology

All tissue specimens had been fixed in formalin and were embedded in paraffin (FFPE). Deparaffinized tissue sections were stained with hematoxylin and eosin. Histological re-examination of primary tissue sections was carried out for all cases to assure if inclusion criteria were met. The tumors were classified according to Lauren^2^. pTNM-stage of all patients was determined according to the 8th edition of the UICC guidelines and was based on surgical pathological examinations^17^.

### Assessment of further clinicopathological characteristics

The assessment of the αvβ3 and αvβ5 integrin^18^, claudin-18.2—^19^, HER2—^20^, *KRAS*—^16^, MET—^21^, p53—^22^, PD-L1—^23^ and *PIK3CA* status^24^ was performed as described in detail elsewhere. Infection with H. pylori was evaluated histologically, using Giemsa staining and molecular pathologically by polymerase chain reaction^16^. The microsatellite instability (MSI) status was assessed by immunohistochemistry using antibodies directed against MLH1, PMS2, MSH2, and MSH6. For each case with reduced or absent staining, subsequent molecular comparison of the allelic profiles of the mononucleotide repeat markers BAT-25, BAT-26, NR-21, NR-24, and NR-27 in tumor and corresponding normal tissue was carried out^25^. Members of the EpCAM signaling pathway (EpEX, EpICD, β-catenin, ADAM17, PSEN2 and E-Cadherin) had been assessed by immunohistochemistry^26^. Epstein-Barr virus-encoded RNA was detected using the EBER probe (Novocastra) and BondMax detection system according to manufacturer´s instructions (Leica Microbiosystems GmbH)^24^.

### Immunohistochemical detection of RNF43 and LRP1B

Immunohistochemical staining was performed on tissue sections of the primary tumor using the Bondmax Autostainer (Leica Biosystems, Germany) and polyclonal antibodies directed against RNF43 (ab 217787, Abcam, Cambridge, UK) and LRP1B (HPA069094, Sigma-Aldrich, St. Louis, MO, USA) in 1:100 dilutions^27,28^. All tissue sections were pretreated for 20 min with ER1-antigen retrieval solution (Leica Biosystems, Germany). For visualization the Bond Polymer Refine Detection Kit DAB (Leica Biosystems, Germany) was used. Counterstaining was performed with hematoxylin.

### Evaluation of RNF43 and LRP1B immunostaining

**Figure 2:**
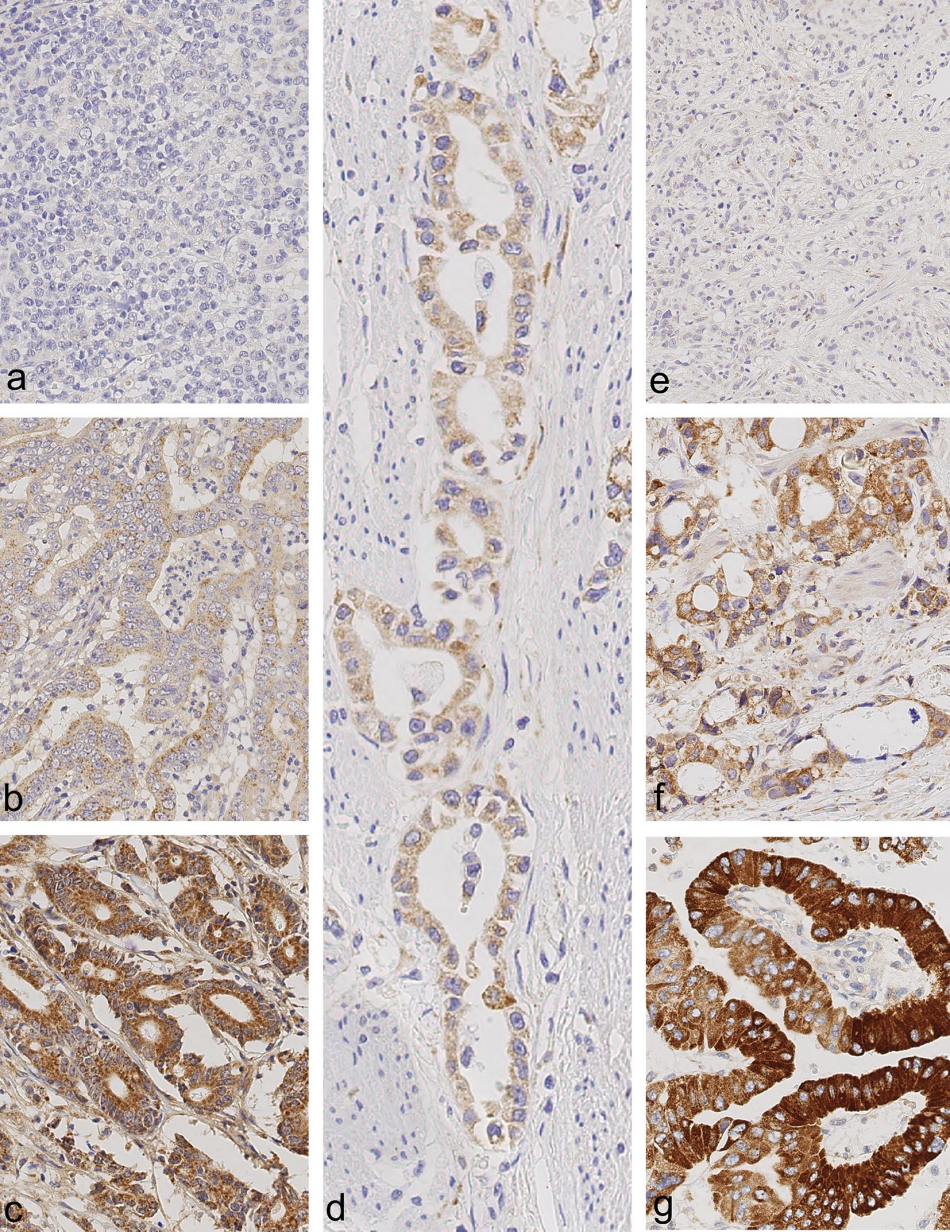
Reference slides for RNF43 (**a**–**c**) and LRP1B (**d**–**g**) immunostaining in gastric cancer. Staining intensities of RNF43 ranged from 0 (**a**; negative) over 1+ (**b**; weak staining) to 2+ (**c**; moderate staining). Images of LRP1B display H-score of 0 (**e**, negative), 1+ (**d**, weak staining), 2+ (**f**; moderate staining) and 3+ (**g**, strong staining). Anti-RNF43-immunostaining and anti-LRP1B-immunostaining, hematoxylin counterstain. Original magnifications ×400.

Cytoplasmatic expression of RNF43 and LRP1B was evaluated using the histoscore (H-score), which included two different parameters: the intensity of immunostaining and the distribution of the stained cells in percentage^29^. The intensity of the immunostaining of LRP1B was categorized into 0 (no staining), 1+ (weak) and 2+ (moderate) and 3+ (strong staining) (Fig. 2). The staining intensities of RNF43 ranged from 0 to 2+ (Fig. 2). The second parameter estimated the percentage of positive tumor cells of each staining intensity [0, 1+, 2+, (3+)] in every single tissue specimen. The sum total of all staining intensities found in a single specimen always added up to a total of 100% tumor cells: % (0) + % (1+) + % (2+) + (% (3+)) = 100%. The H-score was calculated according to the formula: H-score = [0 × percentage of immunonegative tumor cells] + [1 × percentage of weakly stained tumor cells] + [2 × percentage of moderately stained tumor cells] + ([3 × percentage of strongly stained tumor cells]), resulting in a possible H-score between 0 and 300 for LRP1B and between 0 and 200 for RNF43. The maximum possible H-score of LRP1B was 300 if all cells of a tissue specimen showed strong staining: [0 × 0%] + [1 × 0%] + [2 × 0%] + [3 × 100%] = 300. Whereas the maximum possible H-score of RNF43 was 200.

In addition, the patterns of RNF43 and LRP1B staining were noted. Since there is no general guideline to assess heterogeneity in GC, we classified tumors with the same staining intensity across the whole tissue section as “homogeneous” and tumors with different staining intensities especially 3+ and 0 in the same tissue section as “heterogeneous”. “Homogeneous white”, “homogeneous black” and “homogeneous grey” were distinguished. While “homogeneous white” and “homogeneous black” described completely negative or positive GCs for RNF43 or LRP1B expression, tumors classified as “homogeneous grey” showed unstained (0), weakly (1+) and/or moderately (2+) as well as strongly (3+) stained tumor cells intermixed randomly in one and the same tissue section. Tumors with distinct areas of different staining intensities in a single tissue section were classified as “heterogeneous”. Three different heterogeneous staining patterns were distinguished: “black and white”, “black and grey” and “grey and white”, as outlined in detail by Schoop et al.^22^.

### Statistical analysis

Statistical analyses were done using SPSS 27.0 (IBM Corporation, Armonk, New York, USA). The significance of correlation between non-ordinal variables was calculated using the Fisher’s exact test. The Kendall´s tau test was applied for parameters of ordinal scale, e.g., T category, N category and the UICC stage. We assumed a significance level of 0.05. To compensate for the false discovery rate within the correlations, we applied the Simes (Benjamini–Hochberg) procedure (multiple testing correction)^30^. All *p* values are uncorrected. The *p* values, which remained significant after the Simes procedure, are marked in Suppl. Table 1. Median overall survival (OS) and tumor specific survival (TSS) with 95% confidence intervals were determined using the Kaplan–Meier method and differences between survival rates were tested using the log-rank test. Variables having *p* < 0.100 in univariate survival analysis were included in a multivariate survival analysis (Cox regression, backwards LR method).

## Results

The basic clinicopathological characteristics of our validation group are summarized in Table 1 and extended data are shown in Suppl. Table 1. In total, 446 patients fulfilled all study criteria of RNF43 and LRP1B. The age at diagnosis ranged from 28.4 to 92.1 years with a median age of 67.9 years. OS and TSS data were available for 434 (97.3%) and 406 (91.0%) cases. The median OS was 14.7 months and the median TSS was 16.0 months. The median follow-up period was 78.5 months, calculated by Kaplan–Meier estimate of potential follow-up (KM-PF) (reverse Kaplan–Meier)^31^.

**Table 1:** Patient cohort.

	n	(%)
Total	446	(100.0)
*Gender*		
Female	168	(37.7)
Male	278	(62.3)
*Age group*		
< 64 Years	223	(50.0)
≥ 64 Years	223	(50.0)
*Localization*		
Proximal stomach	141	(31.8)
Distal stomach	303	(68.2)
*Lauren phenotype*		
Intestinal	226	(50.7)
Diffuse	140	(31.4)
Mixed	31	(7.0)
Unclassified	49	(11.0)
*Grading (intestinal only)*		
Low	98	(43.4)
High	128	(56.6)
*pT category*		
pT1a/T1b	51	(11.4)
pT2	51	(11.4)
pT3	177	(39.7)
pT4a/pT4b	167	(37.4)
*pN category*		
pN0	124	(27.9)
pN1	62	(13.9)
pN2	81	(18.2)
pN3a/b	178	(40.0)
*pM category*		
pM0	359	(80.5)
pM1	87	(19.5)
*UICC stage*		
IA/B	74	(16.6)
IIA/B	95	(21.3)
IIIA/B/C	189	(42.5)
IV	87	(19.6)
*Lymph node ratio*		
Low (< 0.189)	214	(48.1)
High (≥ 0.189)	231	(51.9)
*pL category*		
L0	206	(48.2)
L1	221	(51.8)
*pV*		
V0	379	(89.0)
V1	47	(11.0)
*pR status*		
pR0	386	(87.3)
pR1/pR2	56	(12.7)
*Overall survival [months]*		
Total/events/censored	434/341/93	
Median Survival	14.7 ± 1.1	
95% confidence interval	12.6–16.7	
*Tumor specific survival [months]*		
Total/events/censored	406/278/128	
Median Survival	16.0 ± 1.3	
95% confidence interval	13.5–18.5	

### Expression of RNF43 and LRP1B in gastric cancer

The expression of RNF43 and LRP1B was studied using whole mount tissue sections.

**Figure 3:**
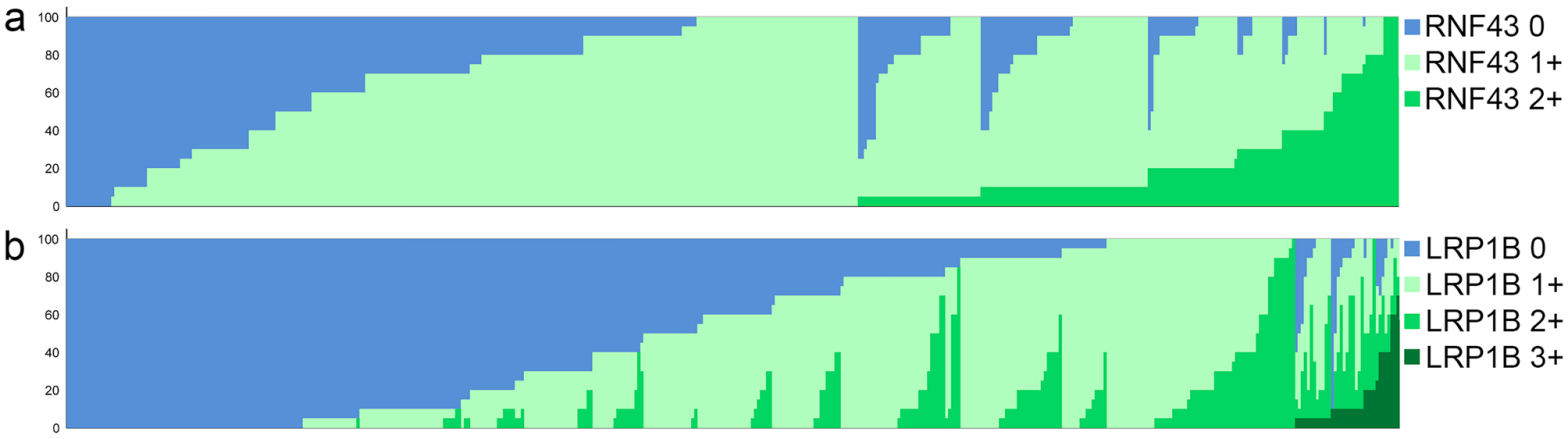
Waterfall-plots illustrating the distribution of RNF43 (**a**) and LRP1B (**b**) immunostaining among 446 patients. The waterfall plots show case-by-case the results of immunohistochemistry (RNF43-IHC and LRP1B-IHC; linear data presentation). The different colours illustrate the different staining intensities: RNF43-IHC 0 (blue), RNF43-IHC 1+ (light green) and RNF43*-IHC* 2+ (medium green) as well as LRP1B-IHC 0 (blue), LRP1B-IHC 1+ (light green), LRP1B-IHC 2+ (medium green) and LRP1B-IHC 3+ (dark green). The sum total of all staining intensities found in a single case always added to a total H-score of 100% according to the following formulas: P (RNF43-IHC 0) + P (RNF43-IHC 1+) + P (RNF43-IHC 2+) = 100% and P (LRP1B-IHC 0) + P (LRP1B-IHC 1 +) + P (LRP1B-IHC 2+) + P (LRP1B-IHC 3+) = 100%.

Among the 446 cases, 431 (96.6%) showed RNF43 immunostaining, while 15 (3.4%) were completely devoid of any RNF43 expression. The percentage of stained tumor cells ranged from 0 to 100%. Staining intensities of RNF43 ranged from 0 (no staining) over 1+ (weak staining) to 2+ (moderate staining) and were discovered in 302 (67.7%), 426 (95.4%) and 181 (40.6%) cases, respectively (Fig. 3). The H-score ranged from 0 to 200 with a median of 90.

367 (82.3%) cases showed LRP1B immunostaining, while 79 (17.7%) were completely immunonegative. The percentage of stained tumor cells ranged from 0 to 100%. Staining intensities of LRP1B ranged from 0 to 3+ (strong staining). Lack of immunostaining of a fraction of tumor cells was found in 369 (82.7%) cases, weak immunostaining (1+) in 359 (80.5%), moderate (2+) in 183 (41.0%) and strong in 35 (7.8%) (Fig. 3). The H-score ranged from 0 to 250 with a median of 60.

### Expression patterns of RNF43 and LRP1B

To further assess the expression patterns of RNF43 and LRP1B in our cohort, we categorized the immunostaining as “homogeneous” and “heterogeneous”. A complete loss of RNF43 or LRP1B expression, i.e., “homogeneous white”, were found in only 15 (3.4%) and 79 (17.7%) cases, respectively. This shows that complete loss of RNF43 or LRP1B is the exception rather than the rule in GC. To the contrary, only 5 (1.1%) cases were “homogeneous black” for RNF43, and none for LRP1B. The majority of GCs showed a variably reduced expression of RNF43 and LRP1B, i.e., either “homogeneous grey” or heterogeneous. The spectrum of staining intensities ranged from two (RNF43: 63.0%; LRP1B: 49.3%) to three (RNF43: 20.4%; LRP1B: 24.7%) or four (LRP1B: 4.5%) different staining intensities found in a single case (Fig. 3). Collectively, these data provide evidence of a substantial intratumoral heterogeneity of the expression patterns of RNF43 and LRP1B in GC.

### Correlations of RNF43 and LRP1B with clinicopathological patient characteristics

In order to find correlations of RNF43 and LRP1B expression with clinicopathological patients characteristics, we dichotomized our study population at the median H-scores.

Following this dichotomizing, RNF43 (*p* < 0.001) and LRP1B (*p* < 0.001) correlated significantly with the phenotype according to Lauren, where a loss of expression was more commonly observed in diffuse type GC compared with intestinal type GC (Suppl. Table 1). We also included molecules, e.g., of the EpCAM signaling pathway, which were shown to interact with the WNT pathway^26^. A reduced or lost expression of RNF43 was also more commonly observed in poorly differentiated tumors, HER2-negative, FZD7-low, EpICD-low and PS2-low GCs (Suppl. Table 1). Most MSI cases (74%) presented a high expression of RNF43. This is in line with Wang et al., who found an over expression of RNF43 on mRNA level in 4 of 10 MSI cases^32^. A reduced or lost expression of LRP1B was also more commonly found in tumors with a high lymph node ratio, HER2-negative, β-catenin-low, FZD7-low, EpICD-low, and PS2-low GCs.

Next, we explored the correlation of RNF43 and LRP1B expression separately for intestinal and diffuse type GC. Regarding intestinal type GCs, reduced or lost expression of RNF43 was more commonly found in GCs without venous invasion and FZD7-low GCs. Low or lost expression of LRP1B was found in patients without distant metastases. Regarding diffuse-type GCs, no association was found between RNF43 and any clinicopathological patient characteristic. Reduced or lost expression of LRP1B was more common in FZD7-low/negative GCs.

Finally, we studied the correlation between RNF43 and LRP1B. A reduced or lost expression of RNF43 correlated significantly with a reduced or lost expression of LRP1B (*p* < 0.001; Suppl. Table 1). The same association was found for intestinal and diffuse type GCs only, which lost significance after correction for multiple testing.

### Correlation of patient survival with RNF43 and LRP1B expression in different Lauren phenotypes

Next, we investigated the relationship between RNF43 and LRP1B, and OS and TSS, respectively (Suppl. Table 1).

**Figure 4:**
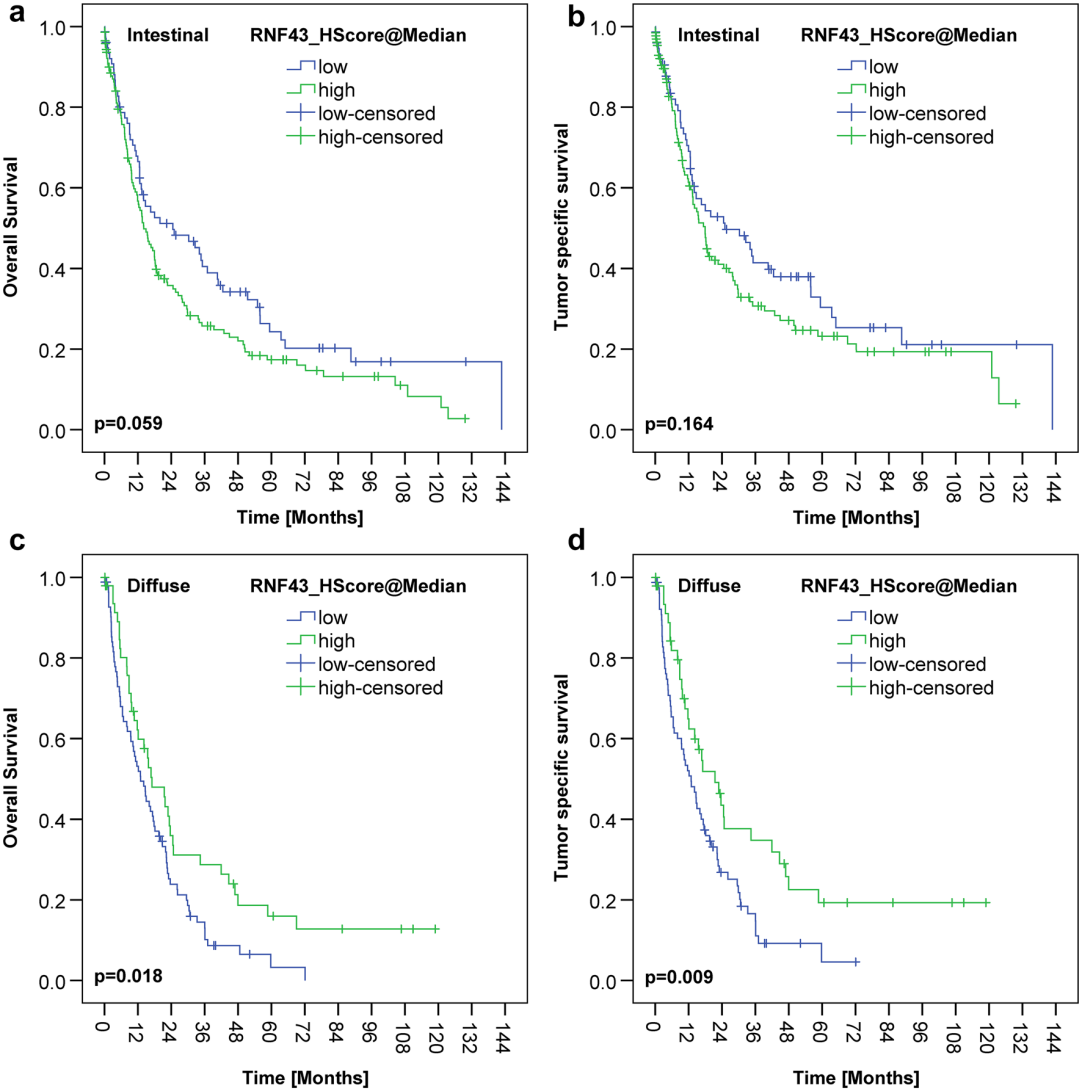
RNF43 and survival depending on Lauren-type. There was no significant correlation with overall or tumor specific survival for either the intestinal (**a**, **b**) or the diffuse gastric cancer (**c**, **d**). However, an interesting trend is that in the intestinal cohort, patients with low RNF43 expression lived longer than those with high RNF43 expression (**a**, **b**). In the diffuse type gastric cancer, patients with high RNF43 expression tended to live slightly longer (**c**, **d**).

Interestingly, differences were noted regarding RNF43 between the intestinal and diffuse type GCs. Survival analysis of the intestinal type GCs showed that RNF43 low/negative GCs tended to have a better outcome compared with RNF43 high/positive GCs [24.5 months OS and 25.0 months TSS vs. 14.1 months OS (*p* = 0.059) and 17.9 months TSS (*p* = 0.164), respectively)]. To the contrary, diffuse type GCs with RNF43 low/negative had a worse outcome compared with RNF43 high/positive GCs [12.9 months OS and 18.2 months TSS vs. 17.1 months OS (*p* = 0.018) and 21.5 months TSS (*p* = 0.009), respectively] (Fig. 4).

Interestingly, on multivariate analysis (Cox regression), RNF43 low/negative versus high/positive was an independent prognosticator of survival in diffuse type GC [hazard ratio (HR) for OS HR = 2.393 and of HR = 2.398 for TSS] (Table 2). In contrast, RNF43 was not an independent predictor of survival in intestinal GCs.

**Table 2:** Independent variables after survival analysis (Cox regression).

	Diffuse type gastric cancer						Intestinal type gastric cancer		
	Overall survival			Tumor specific survival			Overall survival		
	HR	95% CI	*p* Value	HR	95% CI	*p* Value	HR	95% CI	*p* Value
*Stage*			< 0.001			< 0.001			0.002
II versus I	3.290	0.761–14.218	0.111	5.877	0.775–44.590	0.087	2.511	1.411–4.470	0.002
III versus I	7.240	1.745–30.036	0.006	15.231	2.072–112.0	0.007	2.679	1.427–5.030	0.002
IV versus I	9.557	2.225–41.056	0.002	19.337	2.581–144.9	0.004	3.828	1.855–7.900	< 0.001
LNR (high vs. low)							2.149	1.351–3.420	0.001
pV (V1 vs. V0)	2.268	1.014–5.071	0.046						
pR (R1/R2 vs. R0)	3.030	1.731–5.302	< 0.001	3.160	1.811–5.512	< 0.001	2.558	1.406–4.655	0.002
HER2 status (positive vs. negative)	4.238	1.493–12.032	0.007	3.767	1.330–10.670	0.013			
FZD7 status							1.567	1.126–2.180	0.008
RNF43 (low vs. high)	2.393	1.333–3.226	0.001	2.398	1.488–3.861	< 0.001			

Included were all variables having *p* < 0.100 in univariate survival analysis (log-rank test), i.e., UICC stage, lymph node ratio, L category, V category, R status, HER2-status, and RNF43 for diffuse subgroup, and UICC stage, lymph node ratio (LNR), L category, R status, FZD7 status, and RNF43 for intestinal subgroup.

Although loss of RNF43 and/or LRP1B generally reduces survival probability, subtle differences were found with respect to the effect size of RNF43 and LRP1B in the different Lauren phenotypes. Considering the whole cohort, RNF43 showed the strongest impact on survival. While patients with high/positive expression of RNF43 and LRP1B showed a median OS of 17.9 months and a TSS of 22.6 months, patients with RNF43 low/negative and LRP1B high/positive lived the shortest with a median of 10.1 and 11.3 months, respectively. In the intestinal type GCs, LRP1B showed the strongest influence on survival. Likewise, patients with intestinal type GC showed the longest median OS and TSS with 35.2 months when RNF43 and LRP1B were both low/negative. In contrast, the combination of RNF43 high/positive and LRP1B low/negative showed the shortest mean OS of 8.2 months and TSS of 9.6 months. In the diffuse type GCs, RNF43 showed the greatest impact on survival. The median longest survival of 17.1 months was observed in patients whose tumors showed RNF43 high/positive and LRP1B high/positive. RNF43 low/negative in combination with LRP1B high/positive showed the shortest median survival of 6.8 months (Table 3).

**Table 3:** Patient survival depending on the combination of RNF43- and LRP1B expression and tumor type as further detailed in Supplemental Table 1.

	RNF43 × LRP1B	Overall survival [months]			Tumor-specific survival [months]		
			95% CI			95% CI	
		Estimate	Lower bound	Upper bound	Estimate	Lower bound	Upper bound
Whole cohort	RNF43 high/LRP1B high	17.873	13.821	21.924	22.604	14.436	30.771
	RNF43 low/LRP1B low	14.686	10.879	18.493	14.686	10.621	18.751
	RNF43 high/LRP1B low	11.729	7.866	15.592	12.846	7.148	18.544
	RNF43 low/LRP1B high	10.119	5.209	15.029	11.302	5.870	16.734
Intestinal	RNF43 high/LRP1B high	17.281	13.217	21.346	20.271	10.379	30.163
	RNF43 low/LRP1B low	35.154	17.764	52.544	35.154	13.175	57.133
	RNF43 high/LRP1B low	8.246	4.275	12.218	9.626	5.134	14.119
	RNF43 low/LRP1B high	12.583	5.077	20.090	13.240	4.937	21.544
Diffuse	RNF43 high/LRP1B high	17.051	3.130	30.972	17.051	3.130	30.972
	RNF43 low/LRP1B low	14.620	8.877	20.364	14.062	10.432	17.691
	RNF43 high/LRP1B low	16.690	6.931	26.449	23.589	12.575	34.603
	RNF43 low/LRP1B high	6.768	1.810	11.726	6.768	2.496	11.040

Finally, we correlated some combinations of RNF43/LRP1B-expression with clinicopathological patient characteristics. In the intestinal type GC, RNF43 low/negative and LRP1B low/negative correlated significantly with fewer lymph node metastases (*p* = 0.006) and distant metastases (*p* = 0.027), a lower UICC stage (*p* = 0.032), and a lower lymph node ratio (*p* = 0.004) compared with GCs harboring RNF43 high/positive and LRP1B low/negative (Table 3).

### Genotype–phenotype correlation

**Figure 5:**
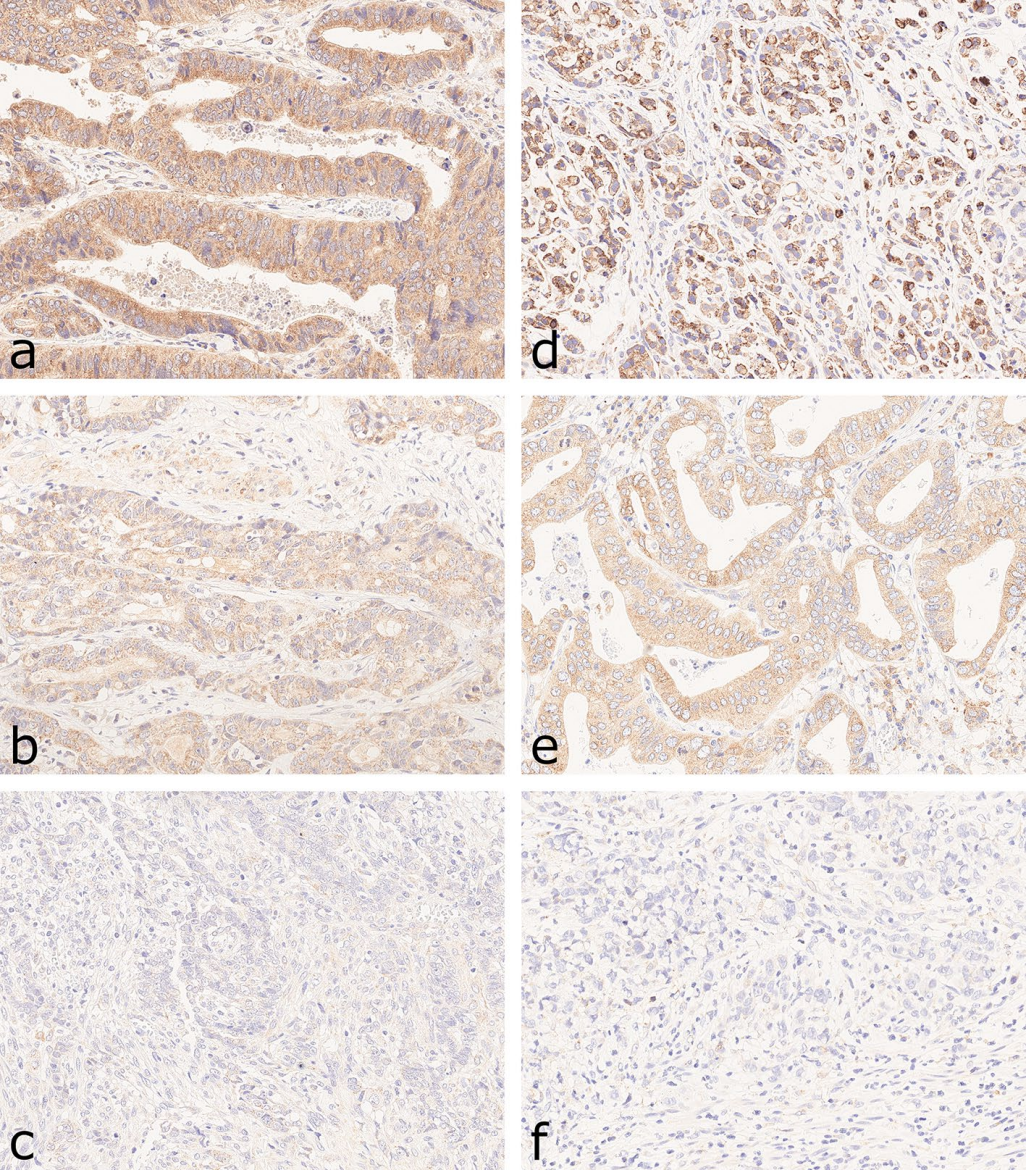
Images of RNF43 (**a**–**c**) and LRP1B (**d**–**f**) immunostaining in gastric cancer for different types of mutations. The wild type shows strong protein expression of RNF43 (**a**) and LRP1B (**d**). The frameshift mutation shows an attenuated protein expression of RNF43 (**b**) and LRP1B (**e**) and the missense mutations shows an almost complete loss of protein expression of RNF43 (**c**) and LRP1B (**f**). Anti-RNF43-immunostaining and anti-LRP1B-immunostaining, hematoxylin counterstain. Original magnifications ×400.

Finally, we correlated genotype and phenotype in nine cases of the discovery cohort, which previously underwent multiregional sequencing (n = 47 tumor samples) (Suppl. Table 2)^4^. The Supplemental Table 2 lists all 9 cases with between 3 and 10 tissue samples per case and mutations (missense, frameshift and nonsense mutation) detected by WES. Of the 9 cases, four had *RNF43* and six *LRP1B* mutations, with several different mutations detectable within a single case as evidence of genetic intratumoral heterogeneity. In order to correlate genotype with phenotype, we performed immunohistochemical staining of 47 paraffin blocks, which covered the anatomical regions of the tumors from which tissue samples were obtained for WES. Histoscore was determined for each paraffin block. For RNF43, the mean histoscore for the validation group was 106 and for LRP1B 86. Interestingly, histoscore varied block wise for both, RNF43 and LRP1B compatible with intratumoral heterogeneity on the phenotypic level. Furthermore, there was a marked loss of protein expression for RNF43 and LRP1B in cases with missense mutations. In contrast, the wild type showed significantly greater protein expression for both. The frameshift mutation showed reduced protein expression compared with the wild type, but no complete loss of expression (Fig. 5).

## Discussion

The comprehensive molecular characterization of GC provided insights into its cancer genome. *RNF43* and *LRP1B* were among the genes, most commonly mutated in GC, lending support to the hypothesis that these genes are important for cancer development and/or progression. Both genes are considered as tumor suppressor genes. They belong to the Wnt signaling pathway, which is one of the most commonly altered pathways in cancer biology in general, and in GC in particular. Tumor suppressors play a crucial role in the initiation, development, and progression of cancer and dysregulation of the Wnt signaling pathway in GC is a major driver and may effect protein expression through regulatory networks (Fig. 1)^5^.

In this study on a large and well-characterized cohort of GC patients, we explored the putative tumor biological significance of RNF43 and LRP1B. Both were expressed by tumor cells in variable amounts and we also found evidence of intra- and intertumoral heterogeneity of their expression patterns. Reduced or loss of expression was significantly associated with each other and with other members of the Wnt signaling pathway, i.e., FZD7 and β-catenin, in that low or lost expression of RNF43 was associated with a low or lost expression of LRP1B. Interestingly, the prognostic significance of RNF43 and LRP1B was different in intestinal and diffuse type GC and its effect on GC biology seems to be context-dependent.

### Intra- and intertumoral heterogeneity of RNF43 and LRP1B expression in gastric cancer

Many previous studies on the same cohort demonstrated a substantial intra- and intertumoral heterogeneity in GC for diverse biomarkers, which stems either form intra- and intertumoral genetic heterogeneity or cancer cell plasticity^20,21,24,33^. Our study demonstrated that this heterogeneity also applies to RNF43 and LRP1B. The majority (83.4% and 78.5%, respectively) of our cases showed at least two different staining intensities for RNF43 and LRP1B, respectively.

There are several putative explanations for the intra- and intertumoral heterogeneity in GC. Multiregional sequencing recently demonstrated that mutations of *RNF43* and *LRP1B* can be clonal and subclonal^4^. Subclonal mutations contribute to intratumoral genetic heterogeneity and hence divergent histological phenotypes. A single case harbored four different mutations of *LRP1B* (case #6; Suppl. Table 2)^4^.

Apart from intratumoral genetic heterogeneity, *RNF43* and *LRP1B* also showed substantial intertumoral heterogeneity in GC: The cosmic database lists 512 cases tested for *RNF43*, of which 144 (28.2%) were mutated, harboring 171 different mutations. Of 633 cases tested for *LRP1B,* 469 (74.1%) were mutated harboring 1320 different mutations^34^. These data underscore the enormous intra- and intertumoral genetic heterogeneity of *RNF43* and *LRP1B*. The multiple distinct genotypes arising from different mutations of *RNF43* and *LRP1B* give rise to the subclonal architecture and shape intratumoral heterogeneity (Suppl. Table 2). However, given the putatively high prevalence of *RNF43* and *LRP1B* mutations in GC, this obviously does not translate into a complete loss of expression. In addition, not all mutations are biologically relevant. Rather, various contextual factors must be taken into account. Mutated *RNF43* and *LRP1B* might be transcribed and translated: it was shown that the recurrent mutation of *RNF43* (p.G659Vfs*41), present in case #6 (Suppl. Table 2), is fully functional^6^. Thus, focusing on mutations only may be misleading and the analysis of expression patterns and tumoral context (i.e., intestinal vs. diffuse type) provide valuable additional information.

Intratumoral phenotypical heterogeneity may also stem from differences in gene expression among different tumor subclones. The spectrum of expression patterns (staining intensities and percentage of stained tumor cells) was highly variable in our cohort for both, RNF43 and LRP1B, rarely showing a clear-cut black and white pattern, as might have been expected, when all would have been related to (subclonal) gene mutations.

According to Knudson-Two-Hit model, both alleles of a tumor suppressor need to be inactivated, e.g., by mutation, loss of heterozygosity (LOH) or epigenetic silencing, to cause a phenotypic change. Our validation group provided evidence that missense mutations were associated with a loss of protein expression of both, RNF43 and LRP1B, respectively. This finding supports the notion that there is a link between genotype and phenotype. As we did not examine LOH and methylation status in our discovery cohort, we cannot exclude that the second alleles were still functional. However, the discovery cohort supported the presence of intratumoral heterogeneity on the genetic and phenotypic level.

Finally, we validated our initial hypothesis of a complex heterogeneous expression of RNF43 and LRP1B in GC. Both seem to be unsuitable as simple prognostic or predictive biomarkers.

### RNF43 and LRP1B expression in gastric cancer are context-dependent

The expression of RNF43 and LRP1B correlated significantly with tumor type according to Lauren, i.e., both were significantly reduced in diffuse type GC, adding them to an increasing list of WNT pathway components, which are differentially mutated and expressed in diffuse and intestinal type GC. Previously we have demonstrated that the expression of E-cadherin, β-catenin, and FZD7, other members of the WNT signaling pathway, is also decreased in diffuse type GC^35^. In addition, diffuse type GCs, more commonly harbor *CDH1* mutations^36^. Collectively, these data illustrate that (1) the dysregulation of the WNT signaling pathway differs between these two types of GC, (2) that the mutation and expression patterns of different Wnt pathway components are interdependent and that (3) they need to be studied separately for intestinal and diffuse type GC, when it comes to correlative analyses with clinicopathological patient characteristics.

In support of this contention, in our series, the correlation between RNF43-expression and patient survival differed between the two tumor types. Patients with intestinal type GC and RNF43 low/negative lived longer (median OS 24.5 months) compared with RNF43 high/positive intestinal type GCs (median OS 14.1 months). Since RNF43 negatively regulates the Wnt signaling pathway as a tumor suppressor, we would have expected that high expression in intestinal type GC would be associated with a less active Wnt signaling pathway and better survival compared with the diffuse type GC. If tumor cells express more RNF43, the Wnt signaling pathway should be less active, since it is regulated by RNF43 via negative feedback mechanism. RNF43, through ubiquitinylation of FZD, causes endocytosis and degradation of the Wnt receptor, leading to loss of Wnt signaling (Fig. 1)^14^. However, these expectations only seem to apply for diffuse type GC, where a decreased or lost expression was associated with a worse outcome (median OS 12.9 months) compared with RNF43 high/positive cases (median OS 17.1 months) (Suppl. Table 1). On multivariate analysis, RNF43 low/negative versus high/positive was even an independent prognosticator of survival in diffuse type GC, supporting its significant role in tumor biology.

Matters become even more complicated, when different combinations of RNF43 and LRP1B-statuses are considered (Table 2). The best outcome was found in intestinal type GCs with RNF43low/LRP1Blow (median OS 35.2 months) and the worst outcome was found in diffuse type GC with the combination of RNF43low/LRP1Bhigh (median OS 6.7 months). Considering RNF43 and LRP1B as tumor suppressors only, does not seem to be sufficient. The putative tumor biological effect is a function of tumor type and the interplay between the diverse members of the Wnt signaling pathway (Fig. 1). Both need to be considered in future studies.

## Conclusions

Summing up, our study is the first extended investigation of the expression patterns of RNF43 and LRP1B in a large and well characterized patient cohort of therapy-naive GCs. While complete loss of expression was the exception, marked intra- and intertumoral heterogeneity of the expression patterns was noted, which likely does not only stem form genetic heterogeneity. Complex regulatory networks of the Wnt-signaling seem to modulate the expression patterns and hence the tumor biological significance, which than also depended on the tumor type. RNF43 and LRP1B are not easy to use prognostic or predictive biomarkers in GC and might require the simultaneous analysis of several members of the Wnt signaling pathway in relation to histological tumor type.

## Supplementary Information

Supplementary Information 1.

Supplementary Information 2.

## Data Availability

The datasets used and/or analysed during the current study are available from the corresponding author on reasonable request.

## Abbreviations

DC: Destruction complex
GC: Gastric cancer
OS: Overall survival
TSS: Tumor-specific survival
