# Supplementary material for: The tumor biological significance of RNF43 and LRP1B in gastric cancer is complex and context-dependent
Source: Sci Rep. 2023 Feb 23;13:3191. doi: 10.1038/s41598-023-30294-8 (PMC9950470; doi:10.1038/s41598-023-30294-8)
Supplement: Supplementary file 1 — Supplementary Information 1. [file 41598_2023_30294_MOESM1_ESM.docx]

**Suppl. Table 1:** Correlations of RNF43 and LRP1B expression in primary resected gastric cancer with clinicopathological patient characteristics. P values were obtained via (a) Fisher’s exact test, (b) Kendall’s tau test, and (c) log-rank test. (d) Significant after FDR-correction.

|  |  |  |  | **Whole Cohort** | | | | | | | | **Intestinal type gastric cancer** | | | | | | | | **Diffuse type gastric cancer** | | | | | | | |
| --- | --- | --- | --- | --- | --- | --- | --- | --- | --- | --- | --- | --- | --- | --- | --- | --- | --- | --- | --- | --- | --- | --- | --- | --- | --- | --- | --- |
|  |  |  |  | **RNF43 Expression** | | | | **LRP1B Expression** | | | | **RNF43 Expression** | | | | **LRP1B Expression** | | | | **RNF43 Expression** | | | | **LRP1B Expression** | | | |
|  |  | **Total** | | **low  (H-score<90)** | | **high  (H-score≥90)** | | **low  (H-score≤60)** | | **high  (H-score>60)** | | **low  (H-score<90)** | | **high  (H-score≥90)** | | **low  (H-score≤60)** | | **high  (H-score>60)** | | **low  (H-score<90)** | | **high  (H-score≥90)** | | **low  (H-score≤60)** | | **high  (H-score>60)** | |
|  |  | **n** | **(%)** | **n** | **(%)** | **n** | **(%)** | **n** | **(%)** | **n** | **(%)** | **n** | **(%)** | **n** | **(%)** | **n** | **(%)** | **n** | **(%)** | **n** | **(%)** | **n** | **(%)** | **n** | **(%)** | **n** | **(%)** |
| **Total** |  |  |  |  |  |  |  |  |  |  |  |  |  |  |  |  |  |  |  |  |  |  |  |  |  |  |  |
|  |  | 446 | (100.0) |  |  |  |  |  |  |  |  |  |  |  |  |  |  |  |  |  |  |  |  |  |  |  |  |
| **Gender** | **n p^(a)^** |  |  | 446 |  |  | 0.079 | 446 |  |  | 0.204 | 226 |  |  | 1.000 | 226 |  |  | 0.878 | 140 |  |  | 0.860 | 140 |  |  | 0.852 |
| Female |  | 168 | (37.7) | 87 | (51.8) | 81 | (48.2) | 93 | (55.4) | 75 | (44.6) | 22 | (35.5) | 40 | (64.5) | 22 | (35.5) | 40 | (64.5) | 50 | (62.5) | 30 | (37.5) | 55 | (68.8) | 25 | (31.3) |
| Male |  | 278 | (62.3) | 120 | (43.2) | 158 | (56.8) | 136 | (48.9) | 142 | (51.1) | 57 | (34.8) | 107 | (65.2) | 62 | (37.8) | 102 | (62.2) | 39 | (65.0) | 21 | (35.0) | 43 | (71.7) | 17 | (28.3) |
| **Age Group** | **n p^(a)^** |  |  | 446 |  |  | 1.000 | 446 |  |  | 0.343 | 226 |  |  | 0.320 | 226 |  |  | 1.000 | 140 |  |  | 1.000 | 140 |  |  | 0.564 |
| < 64 Years |  | 223 | (50.0) | 103 | (46.2) | 120 | (53.8) | 120 | (53.8) | 103 | (46.2) | 28 | (30.8) | 63 | (69.2) | 34 | (37.4) | 57 | (62.6) | 57 | (63.3) | 33 | (36.0) | 61 | (67.8) | 29 | (32.2) |
| >= 64 Years |  | 223 | (50.0) | 104 | (46.6) | 119 | (53.4) | 109 | (48.9) | 114 | (51.1) | 51 | (37.8) | 84 | (62.2) | 50 | (37.0) | 85 | (63.0) | 32 | (64.0) | 18 | (36.4) | 37 | (74.0) | 13 | (26.0) |
| **Localization** | **n p^(a)^** |  |  | 444 |  |  | 0.760 | 444 |  |  | 0.476 | 225 |  |  | 0.262 | 225 |  |  | 1.000 | 139 |  |  | 0.200 | 139 |  |  | 0.272 |
| Proximal stomach |  | 141 | (31.8) | 67 | (47.5) | 74 | (52.5) | 69 | (48.9) | 72 | (51.1) | 37 | (39.4) | 57 | (60.6) | 35 | (37.2) | 49 | (62.8) | 14 | (77.8) | 4 | (22.2) | 15 | (83.3) | 3 | (16.7) |
| Distal stomach |  | 303 | (68.2) | 139 | (45.9) | 164 | (54.1) | 160 | (52.8) | 143 | (47.2) | 42 | (32.1) | 89 | (67.9) | 49 | (37.4) | 82 | (62.6) | 74 | (61.2) | 47 | (38.8) | 83 | (68.6) | 38 | (31.4) |
| **Lauren phenotype** | **n p^(a)^** |  |  | 446 |  |  | <0.001^d^ | 446 |  |  | <0.001^d^ |  |  |  |  |  |  |  |  |  |  |  |  |  |  |  |  |
| Intestinal |  | 226 | (50.7) | 79 | (35.0) | 147 | (65.0) | 84 | (37.2) | 142 | (62.8) |  |  |  |  |  |  |  |  |  |  |  |  |  |  |  |  |
| Diffuse |  | 140 | (31.4) | 89 | (63.6) | 51 | (36.4) | 98 | (70.0) | 42 | (30.0) |  |  |  |  |  |  |  |  |  |  |  |  |  |  |  |  |
| Mixed |  | 31 | (7.0) | 19 | (61.3) | 12 | (38.7) | 22 | (71.0) | 9 | (29.0) |  |  |  |  |  |  |  |  |  |  |  |  |  |  |  |  |
| Unclassified |  | 49 | (11.0) | 20 | (40.8) | 29 | (59.2) | 25 | (51.0) | 24 | (49.0) |  |  |  |  |  |  |  |  |  |  |  |  |  |  |  |  |
| **Grading** | **n p^(a)^** |  |  |  |  |  |  |  |  |  |  | 226 |  |  | 0.575 | 226 |  |  | 0.405 |  |  |  |  |  |  |  |  |
| Low |  | 98 | (43.4) |  |  |  |  |  |  |  |  | 32 | (32.7) | 66 | (67.3) | 33 | (33.7) | 65 | (66.3) |  |  |  |  |  |  |  |  |
| High |  | 128 | (56.6) |  |  |  |  |  |  |  |  | 47 | (36.7) | 81 | (63.3) | 51 | (39.8) | 77 | (60.2) |  |  |  |  |  |  |  |  |
| **pT category** | **n p^(b)^** |  |  | 446 |  |  | 0.373 | 446 |  |  | 0.170 | 226 |  |  | 0.294 | 226 |  |  | 0.976 | 140 |  |  | 0.450 | 140 |  |  | 0.613 |
| T1a / T1b |  | 51 | (11.4) | 24 | (47.1) | 27 | (52.9) | 26 | (51.0) | 25 | (49.0) | 16 | (42.1) | 22 | (57.9) | 15 | (39.5) | 23 | (60.5) | 6 | (54.5) | 5 | (45.5) | 9 | (81.8) | 2 | (18.2) |
| pT2 |  | 51 | (11.4) | 20 | (39.2) | 31 | (60.8) | 22 | (43.1) | 29 | (56.9) | 9 | (29.0) | 22 | (71.0) | 11 | (35.5) | 20 | (64.5) | 7 | (63.6) | 4 | (36.4) | 7 | (63.6) | 4 | (36.4) |
| pT3 |  | 177 | (39.7) | 81 | (45.8) | 96 | (54.2) | 88 | (49.7) | 89 | (50.3) | 35 | (38.5) | 56 | (61.5) | 33 | (36.3) | 58 | (63.7) | 32 | (61.5) | 20 | (38.5) | 37 | (71.2) | 15 | (28.8) |
| pT4a / T4b |  | 167 | (37.4) | 82 | (49.1) | 85 | (50.9) | 93 | (55.7) | 74 | (44.3) | 19 | (28.8) | 47 | (71.2) | 25 | (37.9) | 41 | (62.1) | 44 | (66.7) | 22 | (33.3) | 45 | (68.2) | 21 | (31.8) |
| **pN category** | **n p^(b)^** |  |  | 445 |  |  | 0.403 | 445 |  |  | 0.098 | 226 |  |  | 0.237 | 226 |  |  | 0.605 | 140 |  |  | 0.123 | 140 |  |  | 0.754 |
| pN0 |  | 124 | (27.9) | 56 | (45.2) | 68 | (54.8) | 57 | (46.0) | 67 | (54.0) | 33 | (43.4) | 43 | (56.6) | 26 | (34.2) | 50 | (65.8) | 18 | (54.5) | 15 | (45.5) | 25 | (75.8) | 8 | (24.2) |
| pN1 |  | 62 | (13.9) | 27 | (43.5) | 35 | (56.5) | 31 | (50.0) | 31 | (50.0) | 10 | (31.3) | 22 | (68.8) | 13 | (40.6) | 19 | (59.4) | 13 | (61.9) | 8 | (38.1) | 14 | (66.7) | 7 | (33.3) |
| pN2 |  | 81 | (18.2) | 35 | (43.2) | 46 | (56.8) | 41 | (50.6) | 40 | (49.4) | 8 | (21.1) | 30 | (78.9) | 14 | (36.8) | 24 | (63.2) | 15 | (60.0) | 10 | (40.0) | 16 | (64.0) | 9 | (36.0) |
| pN3a/b |  | 178 | (40.0) | 88 | (49.4) | 90 | (50.6) | 99 | (55.6) | 79 | (44.4) | 28 | (35.0) | 52 | (65.0) | 31 | (38.8) | 49 | (61.3) | 43 | (70.5) | 18 | (29.5) | 43 | (70.5) | 18 | (29.5) |
| **pM category** | **n p^(a)^** |  |  | 446 |  |  | 0.811 | 446 |  |  | 0.551 | 226 |  |  | 0.067 | 226 |  |  | **0.029** | 140 |  |  | 0.844 | 140 |  |  | 0.531 |
| pM0 |  | 359 | (80.5) | 168 | (46.8) | 191 | (53.2) | 187 | (52.1) | 172 | (47.9) | 73 | (37.4) | 122 | (62.6) | 78 | (40.0) | 117 | (60.0) | 66 | (64.1) | 37 | (35.9) | 74 | (71.8) | 29 | (28.2) |
| pM1 |  | 87 | (19.5) | 39 | (44.8) | 48 | (55.2) | 42 | (48.3) | 45 | (51.7) | 6 | (19.4) | 25 | (80.6) | 6 | (19.4) | 25 | (80.6) | 23 | (62.2) | 14 | (37.8) | 24 | (64.9) | 13 | (35.1) |
| **UICC Stage** | **n p^(b)^** |  |  | 445 |  |  | 0.935 | 445 |  |  | 0.331 | 226 |  |  | 0.063 | 226 |  |  | 0.559 | 140 |  |  | 0.750 | 140 |  |  | 0.407 |
| IA/B |  | 74 | (16.6) | 33 | (44.6) | 41 | (55.4) | 35 | (47.3) | 39 | (52.7) | 22 | (41.5) | 31 | (58.5) | 20 | (37.7) | 33 | (62.3) | 9 | (56.3) | 7 | (43.8) | 12 | (75.0) | 4 | (25.0) |
| IIA/B |  | 95 | (21.3) | 44 | (46.3) | 51 | (53.7) | 42 | (44.2) | 53 | (55.8) | 19 | (38.0) | 31 | (62.0) | 17 | (34.0) | 33 | (66.0) | 18 | (62.1) | 11 | (37.9) | 21 | (72.4) | 8 | (27.6) |
| IIIA/B/C |  | 189 | (42.5) | 90 | (47.6) | 99 | (52.4) | 109 | (57.7) | 80 | (42.3) | 32 | (34.8) | 60 | (65.2) | 6 | (44.6) | 51 | (55.4) | 39 | (67.2) | 19 | (32.8) | 41 | (70.7) | 17 | (29.3) |
| IV |  | 87 | (19.6) | 39 | (44.8) | 48 | (55.2) | 42 | (48.3) | 45 | (51.7) | 6 | (19.4) | 25 | (80.6) | 84 | (19.4) | 25 | (80.6) | 23 | (62.2) | 14 | (37.8) | 24 | (64.9) | 13 | (35.1) |
| **LN Ratio** | **n p^(a)^** |  |  | 445 |  |  | 0.569 | 445 |  |  | **0.029** | 226 |  |  | 0.327 | 226 |  |  | 0.129 | 140 |  |  | 0.381 | 140 |  |  | 0.578 |
| Low (<0.189) |  | 214 | (48.1) | 96 | (44.9) | 118 | (55.1) | 98 | (45.8) | 116 | (54.2) | 47 | (38.2) | 76 | (61.8) | 40 | (32.5) | 83 | (67.5) | 35 | (59.3) | 24 | (40.7) | 43 | (72.9) | 16 | (27.1) |
| High (≥0.189) |  | 231 | (51.9) | 110 | (47.6) | 121 | (52.4) | 130 | (56.3) | 101 | (43.7) | 32 | (31.1) | 71 | (68.9) | 44 | (42.7) | 59 | (57.3) | 54 | (66.7) | 27 | (33.3) | 55 | (67.9) | 26 | (32.1) |
| **pL category** | **n p^(a)^** |  |  | 427 |  |  | 0.334 | 427 |  |  | 0.699 | 217 |  |  | 0.478 | 217 |  |  | 0.675 | 132 |  |  | 0.147 | 132 |  |  | 0.260 |
| L0 |  | 206 | (48.2) | 91 | (44.2) | 115 | (55.8) | 103 | (50.0) | 103 | (50.0) | 43 | (38.1) | 70 | (61.9) | 41 | (36.3) | 72 | (63.7) | 35 | (56.5) | 27 | (43.5) | 46 | (74.2) | 16 | (25.8) |
| L1 |  | 221 | (51.8) | 108 | (48.9) | 113 | (51.1) | 115 | (52.0) | 106 | (48.0) | 34 | (32.7) | 70 | (67.3) | 41 | (39.4) | 63 | (60.6) | 49 | (70.0) | 21 | (30.0) | 45 | (64.3) | 25 | (35.7) |
| **pV category** | **n p^(a)^** |  |  | 426 |  |  | 0.439 | 426 |  |  | 0.759 | 216 |  |  | **0.020** | 216 |  |  | 1.000 | 132 |  |  | 0.421 | 132 |  |  | 1.000 |
| V0 |  | 379 | (89.0) | 179 | (47.2) | 200 | (52.8) | 195 | (51.5) | 184 | (48.5) | 73 | (37.8) | 120 | (62.2) | 73 | (37.8) | 120 | (62.2) | 78 | (62.4) | 47 | (37.6) | 86 | (68.8) | 39 | (31.2) |
| V1 |  | 47 | (11.0) | 19 | (40.4) | 28 | (59.6) | 23 | (48.9) | 24 | (51.1) | 3 | (13.0) | 20 | (87.0) | 9 | (39.1) | 14 | (60.9) | 6 | (85.7) | 1 | (14.3) | 5 | (71.4) | 2 | (28.6) |
| **R status** | **n p^(1)^** |  |  | 442 |  |  | 0.570 | 442 |  |  | 0.115 | 224 |  |  | 0.605 | 224 |  |  | 0.299 | 138 |  |  | 0.494 | 138 |  |  | 0.232 |
| R0 |  | 386 | (87.3) | 177 | (45.9) | 209 | (54.1) | 205 | (53.1) | 181 | (46.9) | 72 | (34.8) | 135 | (65.2) | 80 | (38.6) | 127 | (61.4) | 73 | (64.6) | 40 | (35.4) | 82 | (72.6) | 31 | (27.4) |
| R1 / R2 |  | 56 | (12.7) | 28 | (50.0) | 28 | (50.0) | 23 | (41.1) | 33 | (58.9) | 7 | (41.2) | 10 | (58.8) | 4 | (23.5) | 13 | (76.5) | 14 | (56.0) | 11 | (44.0) | 15 | (60.0) | 10 | (40.0) |
| **HER2 Status** | **n p^(a)^** |  |  | 419 |  |  | **0.048** | 419 |  |  | **0.021** | 212 |  |  | 1.000 | 212 |  |  | 0.207 | 128 |  |  | 0.060 | 128 |  |  | 0.155 |
| Negative |  | 385 | (91.9) | 184 | (47.8) | 201 | (52.2) | 205 | (53.2) | 180 | (46.8) | 66 | (35.7) | 119 | (64.3) | 73 | (39.5) | 112 | (60.5) | 80 | (65.0) | 43 | (35.0) | 88 | (71.5) | 35 | (28.5) |
| Positive |  | 34 | (8.1) | 10 | (29.4) | 24 | (70.6) | 11 | (32.4) | 23 | (67.6) | 9 | (33.3) | 18 | (66.7) | 7 | (25.9) | 20 | (74.1) | 1 | (20.0) | 4 | (80.0) | 2 | (40.0) | 3 | (60.0) |
| **H. pylori Status** | **n p^(a)^** |  |  | 381 |  |  | 0.777 | 381 |  |  | 0.777 | 191 |  |  | 0.667 | 191 |  |  | 0.677 | 115 |  |  | 0.189 | 115 |  |  | 1.000 |
| Negative |  | 322 | (84.5) | 145 | (45.0) | 177 | (55.0) | 167 | (51.9) | 155 | (48.1) | 57 | (35.0) | 106 | (65.0) | 60 | (36.8) | 103 | (63.2) | 58 | (59.8) | 39 | (40.2) | 71 | (73.2) | 26 | (26.8) |
| Positive |  | 59 | (15.5) | 28 | (47.5) | 31 | (52.5) | 29 | (49.2) | 30 | (50.8) | 8 | (28.6) | 20 | (71.4) | 9 | (32.1) | 19 | (67.9) | 14 | (77.8) | 4 | (22.2) | 13 | (72.2) | 5 | (27.8) |
| **EBV Status** | **n p^(1)^** |  |  | 433 |  |  | 0.816 | 433 |  |  | 0.489 | 222 |  |  | 0.200 | 222 |  |  | 1.000 | 134 |  |  | 0.366 | 134 |  |  | 1.000 |
| Negative |  | 414 | (95.6) | 191 | (46.1) | 223 | (53.9) | 211 | (51.0) | 203 | (49.0) | 72 | (34.1) | 139 | (65.9) | 77 | (36.5) | 134 | (63.5) | 85 | (63.9) | 48 | (36.1) | 91 | (68.4) | 42 | (31.6) |
| Positive |  | 19 | (4.4) | 8 | (42.1) | 11 | (57.9) | 8 | (42.1) | 11 | (57.9) | 6 | (54.5) | 5 | (45.5) | 4 | (36.4) | 7 | (63.6) | 0 | (0.0) | 1 | (100.0) | 1 | (100.0) | 0 | (0.0) |
| **MSI Status** | **n p^(a)^** |  |  | 433 |  |  | **0.013** | 433 |  |  | 0.111 | 220 |  |  | 0.245 | 220 |  |  | 0.648 | 135 |  |  |  | 135 |  |  |  |
| MSS |  | 398 | (91.9) | 191 | (48.0) | 207 | (52.0) | 209 | (52.2) | 189 | (47.5) | 72 | (36.4) | 126 | (63.6) | 75 | (37.9) | 123 | (62.1) | 86 | (63.7) | 49 | (36.3) | 94 | (69.6) | 41 | (30.4) |
| MSI |  | 35 | (8.1) | 9 | (25.7) | 26 | (74.3) | 13 | (37.1) | 22 | (62.9) | 5 | (22.7) | 17 | (77.3) | 7 | (31.8) | 15 | (68.2) | 86 | (63.7) | 49 | (36.3) | 94 | (69.6) | 41 | (30.4) |
| **PIK3CA** | **n p^(a)^** |  |  | 445 |  |  | 0.213 | 445 |  |  | 0.092 | 226 |  |  | 0.584 | 226 |  |  | 0.179 | 139 |  |  | 1.000 | 139 |  |  | 1.000 |
| Wildtype |  | 421 | (94.6) | 198 | (47.0) | 223 | (53.0) | 220 | (52.3) | 201 | (47.7) | 75 | (35.5) | 136 | (64.5) | 81 | (38.4) | 130 | (61.6) | 86 | (63.2) | 50 | (36.8) | 95 | (69.9) | 41 | (30.1) |
| Mutated |  | 24 | (5.4) | 8 | (33.3) | 16 | (66.7) | 8 | (33.3) | 16 | (66.7) | 4 | (26.7) | 11 | (73.3) | 3 | (20.0) | 12 | (80.0) | 2 | (66.7) | 1 | (33.3) | 2 | (66.7) | 1 | (33.3) |
| **E-Cadherin** | **n p^(a)^** |  |  | 414 |  |  | 0.090 | 414 |  |  | 0.737 | 210 |  |  | 0.286 | 210 |  |  | 0.766 | 128 |  |  | 0.599 | 128 |  |  | 1.000 |
| low |  | 308 | (74.4) | 150 | (48.7) | 158 | (51.3) | 160 | (51.9) | 148 | (48.1) | 51 | (37.0) | 87 | (63.0) | 52 | (37.7) | 86 | (62.3) | 70 | (63.6) | 40 | (36.4) | 75 | (68.2) | 35 | (31.8) |
| high |  | 106 | (25.6) | 41 | (38.7) | 65 | (61.3) | 53 | (50.0) | 53 | (50.0) | 21 | (29.2) | 51 | (70.8) | 29 | (40.3) | 43 | (59.7) | 13 | (72.2) | 5 | (27.8) | 13 | (72.2) | 5 | (27.8) |
| **β-Catenin** | **n p^(a)^** |  |  | 416 |  |  | 0.198 | 416 |  |  | **0.006** | 212 |  |  | 0.471 | 212 |  |  | 0.196 | 129 |  |  | 0.299 | 129 |  |  | 0.082 |
| low |  | 237 | (57.0) | 116 | (48.9) | 121 | (51.1) | 134 | (56.5) | 103 | (43.5) | 34 | (38.2) | 55 | (61.8) | 38 | (42.7) | 51 | (57.3) | 63 | (65.6) | 33 | (34.4) | 70 | (72.9) | 26 | (27.1) |
| high |  | 179 | (43.0) | 76 | (42.5) | 103 | (57.5) | 76 | (42.5) | 103 | (57.5) | 41 | (33.3) | 82 | (66.7) | 41 | (33.3) | 82 | (66.7) | 18 | (54.5) | 15 | (45.5) | 18 | (54.5) | 15 | (45.5) |
| **LGR5** | **n p^(a)^** |  |  | 84 |  |  | 1.000 | 84 |  |  | 1.000 | 82 |  |  | 1.000 | 82 |  |  | 0.821 |  |  |  |  |  |  |  |  |
| low |  | 42 | (50.0) | 14 | (33.3) | 28 | (66.7) | 15 | (35.7) | 27 | (64.3) | 13 | (32.5) | 27 | (67.5) | 14 | (35.0) | 26 | (65.0) |  |  |  |  |  |  |  |  |
| high |  | 42 | (50.0) | 13 | (31.0) | 29 | (69.0) | 16 | (38.1) | 26 | (61.9) | 13 | (32.5) | 29 | (69.0) | 16 | (38.1) | 26 | (61.9) |  |  |  |  |  |  |  |  |
| **ADAM17** | **n p^(a)^** |  |  | 420 |  |  | 0.323 | 420 |  |  | 0.743 | 214 |  |  | 0.101 | 214 |  |  | 0.332 | 130 |  |  | 0.564 | 130 |  |  | 0.414 |
| low |  | 305 | (72.6) | 145 | (47.5) | 160 | (52.5) | 158 | (51.8) | 147 | (48.2) | 62 | (38.8) | 98 | (61.3) | 63 | (39.4) | 97 | (60.6) | 57 | (64.0) | 32 | (36.0) | 64 | (71.9) | 25 | (28.1) |
| high |  | 115 | (27.4) | 48 | (41.7) | 67 | (58.3) | 57 | (49.6) | 58 | (50.4) | 14 | (25.9) | 40 | (74.1) | 17 | (31.5) | 37 | (68.5) | 24 | (58.5) | 17 | (41.5) | 26 | (63.4) | 15 | (36.6) |
| **FZD7** | **n p^(a)^** |  |  | 419 |  |  | **0.002** | 419 |  |  | **0.004** | 217 |  |  | **0.015** | 217 |  |  | 0.475 | 129 |  |  | 0.381 | 129 |  |  | **0.041** |
| low |  | 272 | (64.9) | 139 | (51.1) | 133 | (48.9) | 152 | (55.9) | 120 | (44.1) | 54 | (42.2) | 74 | (57.8) | 50 | (39.1) | 78 | (60.9) | 66 | (66.0) | 34 | (34.0) | 73 | (73.0) | 27 | (27.0) |
| high |  | 147 | (35.1) | 52 | (35.4) | 95 | (64.6) | 60 | (40.8) | 87 | (59.2) | 23 | (25.8) | 66 | (74.2) | 30 | (33.7) | 59 | (66.3) | 16 | (55.2) | 13 | (44.8) | 15 | (51.7) | 14 | (48.3) |
| **EpEx** | **n p^(a)^** |  |  | 417 |  |  | 0.238 | 417 |  |  | 0.336 | 214 |  |  | 0.234 | 214 |  |  | 0.555 | 131 |  |  | 0.836 | 131 |  |  | 1.000 |
| low |  | 293 | (70.3) | 143 | (48.8) | 150 | (51.2) | 155 | (52.9) | 138 | (47.1) | 54 | (39.1) | 84 | (60.9) | 54 | (39.1) | 84 | (60.9) | 63 | (64.9) | 34 | (35.1) | 67 | (69.1) | 30 | (30.9) |
| high |  | 124 | (29.7) | 52 | (41.9) | 72 | (58.1) | 59 | (47.6) | 65 | (52.4) | 23 | (30.3) | 53 | (69.7) | 26 | (34.2) | 50 | (65.8) | 21 | (61.8) | 13 | (38.2) | 24 | (70.6) | 10 | (29.4) |
| **EpICD** | **n p^(a)^** |  |  | 419 |  |  | **0.046** | 419 |  |  | **0.005** | 216 |  |  | 0.051 | 216 |  |  | 0.221 | 127 |  |  | 0.581 | 127 |  |  | 0.058 |
| low |  | 134 | (32.0) | 72 | (53.7) | 62 | (46.3) | 82 | (61.2) | 52 | (38.8) | 21 | (48.8) | 22 | (51.2) | 20 | (46.5) | 23 | (53.5) | 41 | (61.2) | 26 | (38.8) | 51 | (76.1) | 16 | (23.9) |
| high |  | 285 | (68.0) | 122 | (42.8) | 163 | (57.2) | 131 | (46.0) | 154 | (54.0) | 56 | (32.4) | 117 | (67.6) | 62 | (35.8) | 111 | (64.2) | 40 | (66.7) | 20 | (33.3) | 36 | (60.0) | 24 | (40.0) |
| **PS2** | **n p^(a)^** |  |  | 360 |  |  | **0.020** | 360 |  |  | **0.006** | 184 |  |  | 0.053 | 184 |  |  | 0.354 | 112 |  |  | 0.182 | 112 |  |  | 0.244 |
| low |  | 187 | (51.9) | 96 | (51.3) | 91 | (48.7) | 112 | (59.9) | 75 | (40.1) | 30 | (42.3) | 41 | (57.7) | 32 | (45.1) | 39 | (54.9) | 51 | (61.4) | 32 | (38.6) | 60 | (72.3) | 23 | (27.7) |
| high |  | 173 | (48.1) | 67 | (38.7) | 106 | (61.3) | 78 | (45.1) | 95 | (54.9) | 31 | (27.4) | 82 | (72.6) | 42 | (37.2) | 71 | (62.8) | 22 | (75.9) | 7 | (24.1) | 17 | (58.6) | 12 | (41.4) |
| **LRP1B** | **n p^(a)^** |  |  | 446 |  |  | <0.001^d^ |  |  |  |  | 226 |  |  | **0.003** |  |  |  |  | 140 |  |  | **0.013** |  |  |  |  |
| low |  | 229 | (51.3) | 135 | (59.0) | 94 | (41.0) |  |  |  |  | 40 | (47.6) | 44 | (52.4) |  |  |  |  | 69 | (70.4) | 29 | (29.6) |  |  |  |  |
| high |  | 217 | (48.7) | 72 | (33.2) | 145 | (66.8) |  |  |  |  | 39 | (27.5) | 103 | (72.5) |  |  |  |  | 20 | (47.6) | 22 | (52.4) |  |  |  |  |
| **Overall Survival [months]** | **p^(c)^** |  |  |  |  |  | 0.278 |  |  |  | 0.183 |  |  |  | 0.59 |  |  |  | 0.874 |  |  |  | **0.018** |  |  |  | 0.652 |
| Total / events / censored |  | 434 / 341 / 93 | | 202 / 166 / 36 | | 232 / 175 / 57 | | 225 / 184 / 41 | | 209 / 157 / 52 | | 77 / 56 / 21 | | 143 / 113 / 30 | | 82 / 65 / 17 | | 138 / 104 / 34 | | 86 / 75 / 11 | | 50 / 37 / 13 | | 96 / 81 / 15 | | 40 / 31 / 9 | |
| Median Survival |  | 14.7 ± 1.1 | | 13.8 ± 1.5 | | 15.5 ± 1.6 | | 13.8 ± 1.3 | | 16.0 ± 1.7 | | 24.5 ± 9.6 | | 14.1 ± 2.0 | | 15.6 ± 3.3 | | 16.0 ± 1.9 | | 12.9 ± 2.1 | | 17.1 ± 3.9 | | 15.5 ± 2.2 | | 12.1 ± 4.4 | |
| 95% C.I. |  | 12.6 - 16.7 | | 10.9 - 16.8 | | 12.4 - 18.5 | | 11.3 - 16.4 | | 12.6 - 19.3 | | 5.6 - 43.4 | | 10.2 - 18.0 | | 9.1 - 22.2 | | 12.3 - 19.7 | | 8.9 - 17.0 | | 9.3 - 24.8 | | 11.1 - 19.8 | | 3.5 - 20.7 | |
| **Tumor Specific Survival [months]** | **p^(c)^** |  |  |  |  |  | 0.082 |  |  |  | 0.145 |  |  |  | 0.164 |  |  |  | 0.785 |  |  |  | **0.009** |  |  |  | 0.719 |
| Total / events / censored |  | 406 / 278 / 128 | | 192 / 143 / 49 | | 214 / 135 / 79 | | 212 / 151 / 61 | | 194 / 127 / 67 | | 75 / 49 / 26 | | 131 / 87 / 44 | | 77 / 52 / 25 | | 129 / 84 / 45 | | 80 / 65 / 15 | | 49 / 31 / 18 | | 90 / 68 / 22 | | 39 / 28 / 11 | |
| Median Survival |  | 16.0 ± 1.3 | | 14.0 ± 1.5 | | 18.2 ± 2.5 | | 14.0 ± 1.5 | | 18.0 ± 1.8 | | 25.0 ± 9.5 | | 17.9 ± 2.1 | | 15.6 ± 3.7 | | 18.2 ± 4.6 | | 18.2 ± 3.4 | | 21.5 ± 4.6 | | 15.0 ± 2.3 | | 14.2 ± 4.3 | |
| 95% C.I. |  | 13.5 - 18.5 | | 11.2 - 16.9 | | 13.3 - 23.1 | | 11.0 - 17.1 | | 14.5 - 21.4 | | 6.3 - 43.7 | | 13.8 - 22.0 | | 8.3 - 23.0 | | 9.2 - 27.2 | | 8.6 - 17.1 | | 12.4 - 30.6 | | 10.5 - 19.5 | | 5.7 - 22.7 | |
