# Supplementary material for: The tumor biological significance of RNF43 and LRP1B in gastric cancer is complex and context-dependent
Source: Sci Rep. 2023 Feb 23;13:3191. doi: 10.1038/s41598-023-30294-8 (PMC9950470; doi:10.1038/s41598-023-30294-8)
Supplement: Supplementary file 2 — Supplementary Information 2. [file 41598_2023_30294_MOESM2_ESM.docx]

**Suppl. Table 2:** Correlation of genotype and phenotype: shown are the H-scores of LRP1B and RNF43 of 47 tumor samples from 9 patients of the discovery cohort with known mutation status.
